# *Otostegia persica* (Lamiaceae): A review on its ethnopharmacology, phytochemistry, and pharmacology

**Published:** 2014

**Authors:** Zahra Sadeghi, Maryam Akaberi, Jafar Valizadeh

**Affiliations:** 1 *Agricultural Research Center, High Educational complex of Saravan, I. R. Iran *; 2 *Biotechnology Research Center and School of Pharmacy, Mashhad University of Medical Sciences, Mashhad, I. R. Iran *; 3 *Department of Biology, University of Sistan & Baluchestan, Zahedan, I. R. Iran *

**Keywords:** *Otostegia*, *Pharmacology*, *Phytochemistry*, *Toxicity*, *Therapeutics*, *Traditional Medicine*

## Abstract

**Objective:** The current study summarizes the updated information concerning the ethnopharmacology, Phytochemistry, and pharmacology of *Otostegia persica* Boiss. (Lamiaceae), an endemic medicinal plant in south and southeast of Iran.

**Materials and Methods:** Information was collected through bibliographic investigation from scientific journals, books, theses, reports, and electronic search (databases SCOPUS, Google Scholar, Web of Science, and Science Direct). Moreover, documentation from unpublished resources and ethnobotanical surveys has been used. The present review covers the literature available from 2003 to 2013.

**Results:** In traditional systems of medicine, this plant is reputed for treating diabetes, arthritis, gastric discomfort, headache, rheumatism, sedative activities, regulating blood pressure, and hyperlipidemia. Phytochemical screening of active components and mineral element evaluation of this species have been reported. Several types of diterpenoids and flavonols including morin, kaempferol, and quercetin are identified from the plant. Most of the pharmacological activity of this plant resides in its ﬂavonoid fraction which causes antimicrobial and antioxidant activities. Various pharmacological studies on *O. persica* show antimicrobial, antioxidant, anti-inflammatory, anti-diabetic, anti-aphid, and hepatoprotective activities.

**Conclusion:** Being an endemic plant of Iran, this species is an important medicinal herb which can be used for various purposes. This review might be helpful for scientists and researchers to find new chemical entities responsible for its claimed traditional uses and discover new lead compounds for diseases mentioned.

## Introduction

Natural products have provided some of the important life saving drugs used in the armamentarium of modern medicine. However, among estimated 250,000-400,000 plant species, only 6% have been studied for biological activity and 15% have been investigated phytochemically. This shows the need for planned activity-guided Phyto-pharmacological evaluation of herbal drugs (Chaudhary et al., 2012 ▶).

Lamiaceae family is one of the largest and most distinctive families of flowering plants with about 220 genera and almost 4000 species worldwide (Naghibi et al., 2005 ▶; Víctor Benavides, 2010). *Otostegia *genus consists of about 33 species (Table 1) which grows mainly in the Mediterranean region and adjoining Asia Minor (Khan et al., 2009 ▶). In Iran, only three species are available, *Otostegia aucheri, O. michauxi, and O. persica*, of which the last two are endemic to Iran (Ayatollahi et al., 2009 ▶). *Otostegia persica* is called "Golder" locally (Yassa et al., 2005 ▶) and occurs mostly in dry tropical and subtropical habitats of Iran.

This article aims to provide an overview of the chemical constituents present in *O. persica* and ethnobotanical and pharmacological actions of this plant.

**Table 1:** Otostegia species

*Otostegia ambigens*	*Otostegia kaiseri*	*Otostegia olgae*
*Otostegia arabica*	*Otostegia kotschyi*	*Otostegia persica*
*Otostegia aucheri*	*Otostegia limbata*	*Otostegia repanda*
*Otostegia benthamiana*	*Otostegia ongipetiolata*	*Otostegia scariosa*
*Otostegia bucharica*	*Otostegia megastegia*	*Otostegia sinaitica*
*Otostegia ellenbeckii*	*Otostegia michauxii*	*Otostegia somala*
*Otostegia erlangeri*	*Otostegia migiurtiana*	*Otostegia steudneri*
*Otostegia fruticosa*	*Otostegia minuccii*	*Otostegia schimperi*
*Otostegia glabricalyx*	*Otostegia modesta*	*Otostegia tomentosa*
*Otostegia hildebrandtii*	*Otostegia moluccoides*	*Otostegia schennikovii *Scharasch
*Otostegia integrifolia*	*Otostegia nikitinae *Scharasch	*Otostegia sogdiana Kudr*

**Morphological description (botanical** **description** **)**

**Figure 1:**
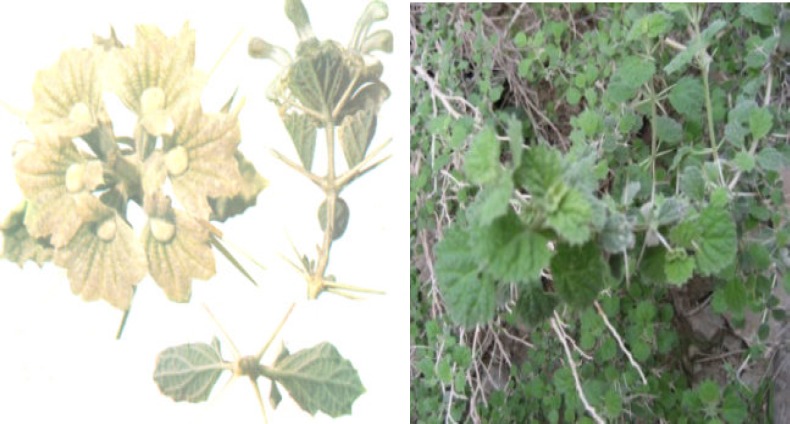
Otostegia persica

*O. persica* is a spiny shrub plant with about 1.5 m height and with rectangular woody stems. Its leaves are opposite on stems with short petiole and obovate blade and covered with dense white hairs. Flowers have funnel-shaped calyx with longitudinal ridges and bilabiate white corolla with hairy upper lip (Figure 1) (Bezenjania et al., 2012 ▶; Recshinger, 1982 ▶).

According to flora of Pakistan, *O. persica* is a shrub with dense glandular stems and suborbicular-obovate, cuneate leaves which have a prominent indumentum of glandular hairs and numerous sessile oil globules. They are creneate to dentate with spines which are present in axils of lower and upper leaves. Spiny bracts are horizontally spreading. Calyx being densely pilose, is 10-nerved or ribbed. Obovoid nutlets are rounded at apex and have oil globules with often only one maturing. (Ghahraman, 1994 ▶; Gahraman, 1996 ▶; Nasir and Ali, 1879 ▶).

**Geographical distribution**

Widely distributed in south and southeast of Iran, *O. persica *Bioss. grows in Fars between Shiraz and Jahrum (southeast of Iran), Kerman, and Sistan & Baluchestan provinces (east region of Iran) (Yassa et al., 2005 ▶; Ghahraman, 1996 ▶). Life forms and chorology of *O. persica* is Irano-Turanian and phanerophyte, respectively (Ghanbarian et al., 2011 ▶)*.*

**Taxonomic Description**

Taxonomic description of the plant is listed in Table 2. 

**Chemical Constituents**

*O. persica* has a range of phytochemical compounds, of which only a few molecules are characterized, so complementary investigations are needed to identify new compounds in this species. Up to now, all investigations have been done on aerial parts of *O. persica,* for instance, Hajhashemi et al. (2004) ▶ reported the presence of flavonoids, steroids, tannins, and triterpenoids in *O. persica*, but up to now all steroids and triterpenoids are not fully reported in this plant. Some known components of the plant are summarized in Table 3 and are as follows:

**Table 2:** Taxonomic description of O. persica

**Systematic classification**	***O. persica***
Kingdom	Plantae
Phylum	Magnoliphyta
Class	Magnoliopsida
Subclass	Asteriddea
Order	Lamiales
Family	Lamiaceae
Genus	*Otostagia*
Species	*Otostegia persica*
Botanical name	*Otostegia persica Bois*
Related Synonym(s)	*Ballota persica (Burm. f.) Benth. *
	*Moluccella persica Burm. f. *
	*Otostegia kotschyi Boiss. *
	*Otostegia microphylla Boiss. *

**Essential Oil**

Sharififar et al.    (2007)  ▶ analyzed the essential oil of flowers and fruits of *O. persica*. Alpha-pinene, 1-octen,3-ol cubenol are the main constituents of the flowers, while diisooctyl phthalate, and hexadecanoic acid are the major components of the essential oil obtained from the fruits. Sadeghi et al also reported caryophyllene oxide, β-sinesal and β-caryophyllene as major constituents of this species. In addition to caryophyllene oxide, Mahmoody identified verbenol in the essential oil. (Mahmoody, 2007 ▶). Geraniol, eugenol, ceryl alcohol, and hentriacontane are present in the essential oil isolated from *O. persica* by Ayatollahi et al. (Ayatollahi et al., 2007 ▶).

**Phenolics**

It has been shown that *O. Persica* contains flavonoids and tannins but it has no alkaloids and saponins (Mahmoody et al., 2007 ▶).

**Figure 2:**
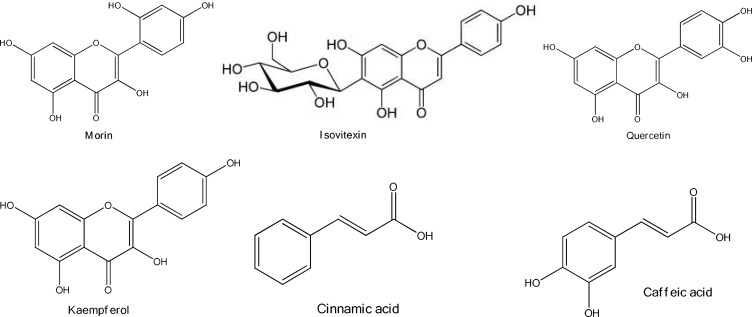
Chemical structure of phenolics found in O. persica

Some of the important constituents of *O. persica *are flavonoids and phenolic compounds including morin, quercetin (Shrififar et al., 2003 ▶), kaempferol, and isovitexin (C-glucoflavone) as well as trans-Cinnamic acid which were identified from methanolic extract of *O. persica* by UV, IR, ^1^H and ^13^C NMR, and MS spectroscopies (Figure 2) (Yassa et al., 2005 ▶). Caffeic acid, ρ-hydroxy benzoic acid, β-sitosterol, and β-sitosteryl acetate were also identified in methanolic extract of *O. persica* (Ayatollahi et al., 2007 ▶).

Alone and in combination with similar phytosterols, β-sitosterol reduces blood levels of cholesterol and is sometimes used in treating hypercholesterolemia. β-sitosterol inhibits cholesterol absorption in the intestine (Matsuoka et al., 2008 ▶).

**Terpenoids**

**Figure3:**
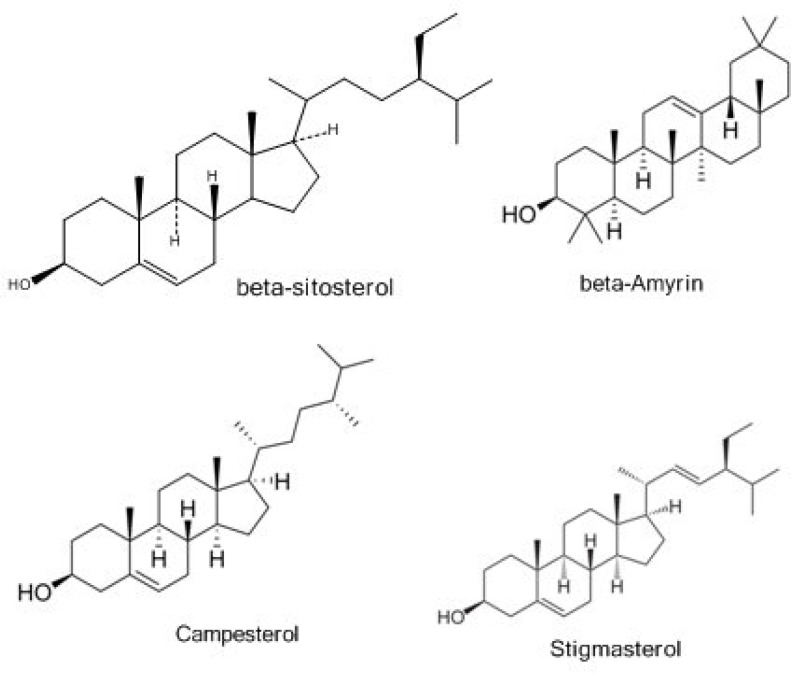
Chemical structures of triterpenes found in O. persica

**Figure 4:**
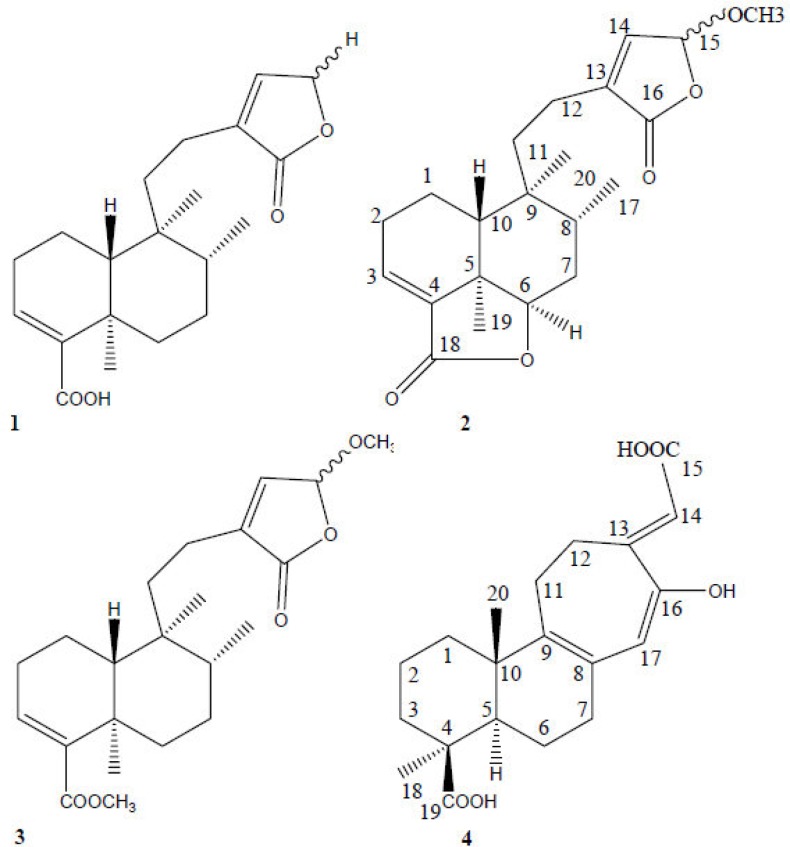
Structure of diterpenes found in O. persica

Diterpenoids are another type of constituents which have been identified from the aerial parts of *Otostegia persica*. Ayatollahi et al. (2007) ▶ showed that dichloromethane extract of aerial parts of *O. persica *could be considered as a rich source of different terpenoids. Triterpene-related compounds such as β-amyrin, campesterol, and stigmasterol are also characterized in this species (Figure 3). Four known diterpenoids belonging to the clerodane and tetracyclic diterpene types were isolated for the first time from* O. persica*. These compounds are known to occur only in genus *Otostegia *(Figure 4). 

**Table 3:** Chemical constituents of O. persica

**Compounds**	**Essential oil**	**Phenolics**	**Terpenoids**	**Minerals**
**Constituents**	alpha-pinene, 1-octen,3-ol cubenol, diisooctyl phthalate, hexadecanoic acid, Caryophyllene oxide, β-sinesal, β- Caryophyllene, verbenol, Geraniol, eugenol, ceryl alcohol, hentriacontane	morin, quercetin, kaempferol, Isovitexin, Trans-Cinnamic acid, Caffeic acid, ρ-hydroxy benzoic acid, β-sitosterol, β-sitosteryl acetate	β-Amyrin, Campesterol, Stigmasterol, New diterpenes	Ca, Mg, Na, Fe, K, Mn, Cr, Cu, Pb and P

**Mineral Composition**

A number of essential minerals are found to be present in *O. persica* (Ayatollahi et al., 2007 ▶). Mineral elements including Ca, Mg, Na, Fe, K, Mn, Cr, Cu, Pb, and P were determined by spectroscopic methods for the first time by Sadeghi (2010) ▶ using two methods of sample preparation: dry ashing and microwave digestion.

**Ethnobotanical uses** **or** **ethnopharmacology**

The importance of *O. persica *is due to its wide variety of medicinal properties. Traditionally, plants belonging to this genus were used for a wide range of medicinal applications. *O. persica *has been widely used by Baluch people, who live in the southeast of Iran. People of this area use the aerial parts of this plant in the forms of decoction and infusion for treatment of headache, diabetes, stomachache, rheumatoid arthritis, toothache, and as anti-hyperlipidemia and analgesic. This species is also used for morphine withdrawal among Baluch people (Sadeghi, 2010 ▶; Javidnia, 2007 ▶, Naghibi et al., 2005 ▶; Sadeghi et al., 2013 ▶).

Safa et al. (2013) ▶ investigated that* O. persica *is commonly used for cardiac distress, reducing palpitation, regulating blood pressure, cough, headache, gastric discomfort, and parasite repellent as well as laxative, carminative, and antipyretic in south of Iran, Hormozgan province.

**Pharmacological activities**

To validate traditional claims associated with the genus, many studies have been carried out using various animal models and *in vitro *assays. These studies show that *Otostegia *species have a potential for developing remedial agents. Some major activities are described below:

**Antimicrobial activity**

Asghari et al. (2006) ▶ evaluated the antimicrobial activities of three extracts of *O. persica* (hexane, followed by chloroform and methanol) against Gram positive and Gram negative strains using well plate, MIC, and MBC methods. *O. persica *extracts showed antimicrobial activity against Gram positive strains including *Listeria monocytogens, Enterococcus fecalis, Staphylococcus aureus*, and *Staphylococcus epidermidis *with MIC values from 0.62 to 20 mg/ml. The MBC values were higher than MIC values for the corresponding MICs. The Gram negative strains, i.e., *Escherichia coli*,* Pseudomonas aeruginosa, Salmonella *spp., *Klebsiella *spp*., *and *Proteus *spp*. *were not inhibited by *O. persica *polar, semi-polar, and non-polar extracts.

Moreover, aqueous and organic extracts of this plant was evaluated for antifungal activity against 2 pathogenic fungus *Aspergillus niger* and *Candida albicans*. It was found that the highest inhibition was obtained with the ethyl acetate extract against *Candida albicans* (Asghari et al., 2006 ▶). Javidnia et al. (2009) ▶ showed that methanolic extract of *O. persica* is effective against specially gram positive bacteria (*S. aureus *and *B. subtilis*). 

**Anti-oxidant activity**

The anti-oxidant activities of different extracts and fractions of aerial parts of *O. persica *were evaluated using beta-carotene bleaching and lipid peroxidation methods (Sharififar et al., 2003 ▶). The inhibitory activities of the plant extracts on the peroxidation of linoleic acid were measured using ferric thiocyanate method in comparison with methanolic extracts of green tea*, Ginkgo biloba*, vitamin E and BHA as positive controls. The results showed that methanolic extract of plant exhibited strong antioxidant activity. More investigation on methanolic extract showed that two compounds were responsible for antioxidant activity. Using UV, IR, MS, and ¹H and ¹³C NMR techniques, it was found that these two compounds were morin and quercetin (Shrififar et al., 2003 ▶ )

The antioxidant activity of the essential oils of *O. persica* was screened using two complementary test systems, i.e., DPPH free radical scavenging and ammonium thiocyanate. In both tested systems, essential oil of the flower exerted greater antioxidant and radical scavenging activity than essential oil of the fruit. In the first test, essential oil of flower exerted antioxidant activity with an IC_50_ 19.8±1.8 μg mL^-1^ almost similar to BHA and ascorbic acid (15.2±1.1 and 17.4±1.3), respectively. In the ammonium thiocyanate system, the inhibition rate of oxidation of linoleic acid for essential oil of flower was estimated 93.5±2.8. The higher activity of this oil in comparison with essential oil of the fruit may be attributed to its high content of monoterpenes, especially oxygenated ones in the oil of the flower (Sharififar et al., 2007 ▶). This biological test for essential oils was also done by Tofighi et al. The IC_50_ of the OSB essential oil was more potent (9.76±1.1) than natural and synthetic antioxidants such as vitamin E (12.02±1.8) and BHA (24.16±2.2) (Tofighi et al., 2009 ▶).

Metanolic extract of *O. persica* consists of flavonoids including morin, kaempferol, quercetin, and Isovitexin which might be responsible for antioxidant activity of the plant. Among these compounds, isovitexin showed the lowest activity which may be due to the absence of a hydroxyl at 3´ position. Comparing antioxidant activity of *O. persica* with *Ginkgo biloba* and green tea shows that its antioxidant potency is more than *Ginkgo biloba* and equal to green tea (Yassa et al., 2005 ▶; ).

**Anti-diabetic effect**

*O. persica *is traditionally used in some regions of Iran as medicinal herb for anti-diabetic properties. Thus, this activity is investigated by some research groups. In 2009, Tofighi et al. investigated anti-diabetic effect of *O. persica*. Anti-diabetic effect of this species on streptozotocin-diabetic rats was also investigated by Ebrahimpoor et al. and Manzari-Tavakoli et al., concluding that this plant had anti-hyperglycemic activity. 

Akbarzadeh et al. (2012) ▶ determined anti-diabetic properties of *O. persica* in STZ- induced diabetic rats and suggested it as a candidate drug for treating diabetes. They proposed that treatment with aqueous extract of *O. persica* would reduce insulin resistance, glucose and triglycerides concentrations, and increase the β-cells regeneration. They contributed this activity to flavonoid quercetin which has a similar effect as metformin, an anti-diabetic drug (Kannappan, 2009 ▶). Some studies show that anti-diabetic effect of quercetin on streptozotocin-induced diabetic rats is related to an increase in insulin absorption and glucose uptake (Cnop et al., 2005 ▶). 

**Anti-glycation activity**

**Figure 5:**
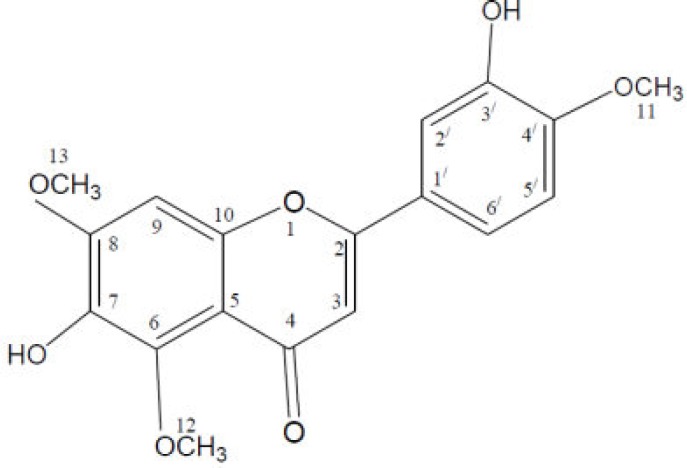
Structure of 3´,7-dihydroxy-4´,6,8-trimethoxy-flavone

Diabetic pathogenesis is accompanied by increased glycation of proteins and accumulation of advanced glycation end products (AGEPs). Glycation and AGEP formation are also associated with the formation of free radicals via autoxidation of glucose and glycated proteins. *O. persica* has anti-glycation activity which is attributed to the presence of 3´,7-dihydroxy-4´,6,8-trimethoxy-flavone (Figure 5). In comparison with standard inhibitor rutin, which shows 83% inhibitory effect, this compound inhibits glycation 65% at 3 mM concentration (; ).

**Anti-** **aphids**

Salari et al. (2010) ▶ determined the effect of acetonic extract of *O. persica* on different pests. The insects were *Aphis fabae *Scopoli, *Aphis gossypii *Glover and *Myzus persicae *(Sulzer) as well as *Tribolium castaneum *(Herbst). The mortality percentage was significantly higher in *A. fabae *and *M. persicae *than in *T. castaneum *treatments.

**Hepatoprotective activity**

The hepatoprotective effect of the methanol extract of aerial parts (shoot) from *O. persica *has been investigated against the carbon tetrachloride (CCl4)-induced acute hepatotoxicity in male rats. Liver damage was assessed using biochemical parameters, i.e., plasma and liver tissue malondialdehyde (MDA), transaminase enzyme levels in plasma (aspartate transaminase (AST) and alanine aminotransferase (ALT)), and liver glutathione (GSH) levels. Results indicate that the methanol extract of *O. persica *shoot is active at 300 mg/Kg (per os) and possesses significant antioxidant and hepatoprotective activities. It is proposed that hepatoprotective mechanisms of this extract on CCl4-induced acute liver damage might be due to the decreased lipid peroxidation (decreased MDA level and increased content of GSH) (Mohammadi et al., 2012).

**Toxicity**

According to SCOPUS, Google Scholar, Web of Science, and Science Direct, no toxicity has been reported for this species.

**Morphine withdrawal**

Effect of *O. persica *on naloxone-induced morphine withdrawal syndrome was studied in male mice. Morphin withdrawal is associated with some signs including jumping, rearing, diarrhea, piloerection, tremor, and ptosis. While oral and i.p. administration of hydroalcoholic extract reduced the number of jumping and rearing, the hexane extract could not exert any significant change. Moreover, the hydroalcoholic extract (1500 mg/kg) significantly (p<0.05) reduced diarrhea, piloerection, tremor, and ptosis. The hexane extract only significantly (p<0.05) inhibited diarrhea. Both oral and i.p. administration of the hydroalcoholic extract reduced the number of jumping episodes in a dose dependent manner. 

Intraperitoneal injection of hydroalcoholic extract was more effective than oral administration. Results of this study indicates that the extract of *O. persica *contains component(s) that alleviate morphine withdrawal syndrome and it is proposed that the responsible constituent(s) is (are) found in polar fraction, since the hexane extract have only a negligible effect. It is also proposed that this activity may be due to the flavonoid components of the plant. However, the active components and the mechanism of action of this plant are not known and further investigations are needed to clarify them (Hajhashemi et al., 2004 ▶).

**Antimalarial activity**

Antimalarial activity of *O. persica* and its combination with chloroquine against both CQ-sensitive and CQ-resistant strains of *Plasmodium berghei *was determined in 2012 by Nateghpour et al. (2012) ▶ via *In-vivo *fixed ratio method. First of all, ED_50_s were calculated. Determination of ED_50_s showed 1.1 mg/Kg and 2.4 mg/Kg of mouse body weight for chloroquine in CQ-sensitive and CQ-resistant strains, respectively, and 450 mg/Kg for *O. persica *in both strains. Results also showed that the combinations of “50% CQ + 50% OP”, “30% CQ + 70% O.P” and “70% CQ + 30% OP” are more effective than other combinations against CQ-sensitive strain. Results suggest that *O. persica *potentiates the effectiveness of chloroquine against the chloroquine-sensitive strain of *P. berghei *but do not affect chloroquine-resistant *P. berghei*.

**Anti-arthritis**

In arthritis, oxidation processes cause lipid peroxidation and formation of low-mass oligosaccharides resulting damage to bone and cartilage. Anti-oxidants are proposed as inhibitors of this process (Bors et al., 1996 ▶) and decrease the inflammation. Studies show that *O. persica*, due to the presence of flavonoids, has strong anti-oxidant activity. As a result, investigations suggest it as an anti-arthritis agent (Yassa et al., 2005 ▶).

**Anti-inflammatory and healing of burn wound**

Methanolic extract of the *O. persica *was newly investigated for accelerating healing process of burn wound. Results show that this extract significantly exhibited healing activity when topically applied on rats. *O. persica* is an effective treatment for saving the burn site (Ganjali et al., 2013 ▶).

**Microscopic analysis**

In 2010, microscopic analysis of aerial parts of *O. persica* was investigated by Tofighi et al. Results showed that *O. persica *powder had a pale yellowish-green color with little odor and bitter taste. The diagnostic characteristics are: (a) the covering trichomes (unicellular and multicellular) with wide base and sometimes warted walls, (b) The epidermis showing diacytic stomata, big cicatrix, spiral, double helix, and annular thickening vessels; some paranchymatous cells had beaded or sinuous walls, (c) The cluster crystals of calcium oxalate, and (d) the epidermal cells of the stigmas which were extended to form long and finger-like papillaes (Tofighi et al., 2010 ▶).

*Otostegia persica* has been explored exhaustively for its phytochemical, pharmacological, and ethnopharmacological activities. From the foregoing accounts, it is evident that *O. persica *plant has been used ethnomedicinally as a valuable therapeutic agent for a variety of diseases, as we have illustrated in this article. Various compounds which exist in this plant may be responsible for its pharmacological activities. More investigations are proposed for determining and discovering compounds responsible for its pharmacological activities. 
